# Genes and brain malformations associated with abnormal neuron positioning

**DOI:** 10.1186/s13041-015-0164-4

**Published:** 2015-11-05

**Authors:** Jeffrey J. Moffat, Minhan Ka, Eui-Man Jung, Woo-Yang Kim

**Affiliations:** Department of Developmental Neuroscience, Munroe-Meyer Institute, University of Nebraska Medical Center, 985960 Nebraska Medical Center, Omaha, NE 68198-5960 USA

**Keywords:** Neuron positioning, Brain malformation, Neuron migration, Lissencephaly, Heterotopia, Polymicrogyria, Microcephaly, Cortical dysplasia, LIS1, DCX, Reelin, TUBA1A

## Abstract

Neuronal positioning is a fundamental process during brain development. Abnormalities in this process cause several types of brain malformations and are linked to neurodevelopmental disorders such as autism, intellectual disability, epilepsy, and schizophrenia. Little is known about the pathogenesis of developmental brain malformations associated with abnormal neuron positioning, which has hindered research into potential treatments. However, recent advances in neurogenetics provide clues to the pathogenesis of aberrant neuronal positioning by identifying causative genes. This may help us form a foundation upon which therapeutic tools can be developed. In this review, we first provide a brief overview of neural development and migration, as they relate to defects in neuronal positioning. We then discuss recent progress in identifying genes and brain malformations associated with aberrant neuronal positioning during human brain development.

## Background

Neuronal positioning is an integral part of the coordinated steps comprising neural circuit formation in embryonic and neonatal development [1]. This process takes place throughout the nervous system at different time points depending on the type of neuron. Although neuronal positioning and migration occurs throughout the central nervous system, we will focus on neuronal positioning in the neocortex of the developing brain. We will present basic information on the process of neuronal positioning and describe the abnormalities that may occur in the human brain. Additionally, genes associated with neuronal positioning abnormalities will be discussed.

Correct positioning of neurons by normal migration plays a critical role in establishing cognitive functions and emotion. Human cognitive activity depends on appropriate brain circuit formation. Disrupted brain wiring due to abnormal neuronal development such as improper neuronal positioning can result in brain malformations, cognitive dysfunction, and seizures [2–4]. The causes of brain malformations associated with positioning and migration defects are varied and include genetic mutations and environmental toxins [1, 5, 6]. Studies of neuronal migration disorders have progressed due to advances in molecular genetics and brain magnetic resonance imaging. The commonly identified disorders of neuronal positioning include lissencephaly and heterotopia [7].

## Neural progenitors as a source of migrating neurons in the human cerebral cortex

Neural progenitors can undergo self-renewal or give rise to neurons at the ventricular/subventricular zone in the developing cerebral cortex [8–10]. Reduced numbers of neural progenitors caused by depletion of progenitor pools or slow proliferation result in microcephaly with otherwise normal brain structure [11, 12]. However, microcephaly can also occur in combination with a migration defect, i.e., microcephaly with pachygyria (Norman-Roberts syndrome) [13]. Thus, the disruptive functions of neural progenitor renewal and neurogenesis may interfere with later developmental aspects such as neuronal migration and positioning in the developing brain.

## Neuronal migration modes

**Fig. 1:**
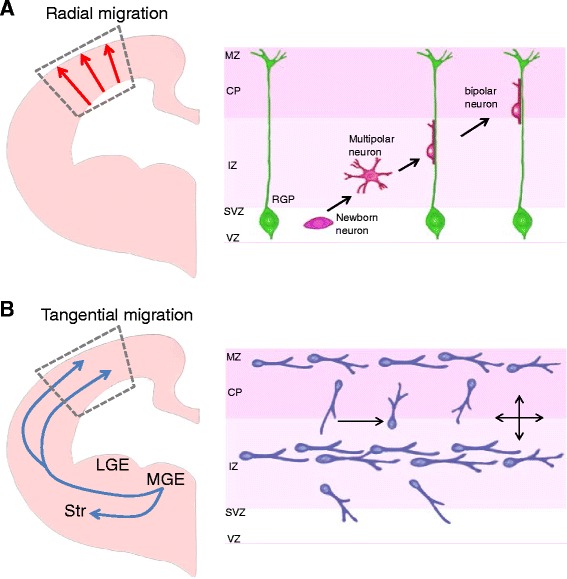
Two modes of neuronal migration in the developing brain. **a** Radial migration. Excitatory pyramidal projection neurons migrate from the ventricular zone to the cortical plate in the developing brain. The right panel shows what happens in the rectangular box in the left panel. Newly-born neurons from radial glial progenitors (RGP) at the ventricular zone (VZ) migrate along the radial processes of RGPs. MZ: marginal zone. CP: cortical plate. IZ: intermediate zone. SVZ: subventricular zone. **b** Tangential migration: Interneurons originate from distinct proliferative zones in the developing brain. Inhibitory interneurons are born in the medial ganglionic eminence (MGE) of ventral brain and migrate in multiple streams into the cerebral wall. Once interneurons reach appropriate spots in the cerebral cortex, they establish their final positions by local adjustment of radial and tangential movement. Unlike pyramidal neurons, these neurons extend multiple leading branches during migration. LGE: lateral ganglionic eminence. LGE: lateral ganglionic eminence. Str: striatum

After neurons are born, they migrate from their birthplaces to their final destinations (Fig. 1). There are two types of embryonic neuronal migration: radial and tangential. The migration of excitatory pyramidal neurons from the cortical ventricular zone (where they are born) is an example of radial migration (Fig. 1a). These neurons migrate into the cortical plate alongside radial glial processes [14–17]. The layers of the cortex form in an “inside-out” manner with later-born pyramidal neurons migrating past earlier-born predecessors in the cortical plate so that they are more superficial in their final position than earlier born neurons [5, 18–20]. In humans, neuronal migration takes place predominantly between 12 and 20 weeks in gestation. The migration of inhibitory interneurons (GABAergic neurons) from the medial ganglionic eminence of the ventral telencephalon (where they are born) is an example of tangential migration (Fig. 1b). Interneurons migrate tangentially to the dorsal telencephalon and then change direction to enter the cortical plate radially [20–23]. Subsets of these cells display ventricle-directed migration followed by radial movement to the cortical plate. Thus, neuronal migration determines the positioning of developing neurons into cortical layers and thereby is important in generating lamina-specific neural circuits. Normal development and function of the neocortex critically depends on the coordinated production and positioning of excitatory and inhibitory neurons [24–27]. Abnormal neuronal migration can arrest different types of neurons at the wrong positions along the migratory path resulting in brain malformations and neurological disorders.

In addition to these well-defined modes of embryonic neuronal migration, a limited number of neurons and neuronal precursors have been shown to migrate and differentiate in the early postnatal rodent and human cerebellum and hypothalamus [28, 29]. Another, more extensive mode of neuronal migration has been observed in adult rodents and non-human primates, in which neuronal precursors migrate along glial projections from the subventricular zone into the olfactory bulbs. This particular passage is referred to as the rostral migratory stream (RMS) [28–30], which continues well into adulthood, but has not been observed in humans [28, 31]. In the RMS, neuronal precursors migrate via a “tunnel” made up of astrocytes into the olfactory bulb, where they then radially migrate in a glial-independent manner toward the glomeruli and differentiate. The majority of these cells eventually become inhibitory neurons, mainly GABAergic granule neurons [28, 32]. Because the application of research tools is currently limited in humans, there is still ongoing debate about whether the RMS exists in humans [28, 31, 32].

In this review we will focus solely on brain malformations thought to be due to abnormal embryonic neuronal migration, although many of the genes and proteins discussed are no doubt involved in both embryonic and postnatal neuronal migration. It is important, however, that further research be done to understand the mechanisms of neuronal migration and the maintenance of neuronal precursor pools in adults, because of the potential to promote regeneration and repair in individuals with neuronal positioning disorders, neurodegenerative disorders, and severe brain injuries.Brain malformations and genes associated with abnormal neuron positioning are listed in Table 1.

**Table 1:** Brain malformations and genes associated with abnormal neuron positioning

Type	Gene	Location	Description
Lissencephaly type I			
Lissencephaly (Autosomal dominant)	LIS1	17p13.3	Microtubule-associated protein
Isolated lissencephaly sequence (ILS) or subcortical band heterotopia (SBH)	TUBA1A	12q13.12	Constituent of microtubules
Miller-Dieker syndrome	LIS1 + YWHAE	17p13.3	Microtubule-associated protein
Lissencephaly (X-linked)			
ILS or SBH	DCX	Xq22.3-q23	Microtubule-associated protein
X-linked lissencephaly with abnormal genitalia	ARX	Xp21.3	Transcription factor
Lissencephaly (Autosomal recessive)			
Lissencephaly with cerebellar hypoplasia (LCH) group b	RELN	7q22	Extracellular matrix serine protease
	VLDLR	9q24	Binds VLDL and transports it into cells by endocytosis
Lissencephaly type II: Cobblestone complex (Autosomal recessive)			
Fukuyama congenital muscular dystrophy or Walker–Warburg syndrome (WWS)	FKTN	9q31.2	Involved in glycosylation
Muscle–eye–brain disease (MEB) or WWS	POMT1	9q34.13	Protein-O-mannosyltransferase 1
	POMT2	14q24.3	Protein-O-mannosyltransferase 2
	POMGNT2	3p22.1	O-linked mannose acetylglucosaminyltransferase
	FKRP	19q13.32	Involved in glycosylation
MEB	LARGE	22q12.3	Glycosyltransferase
	POMGnT1	1p34.1	Participates in O-mannosyl glycosylation
Bilateral frontoparietal polymicrogyria	GPR56	16q21	G protein-coupled receptor 56
CEDNIK syndrome	SNAP29	22q11.21	Synaptosomal-associated protein
Muscular dystrophy	ISPD	7q21.2	Required for protein O-linked mannosylation
	GTDC2	3p22.1	O-linked mannose acetylglucosaminyltransferase
	TMEM5	12q14.2	Glycosyltransferase function
	B3GALNT2	1q42.3	Beta-1,3-N-acetylgalactosaminyltransferase
	SGK196	8q11.21	Protein O-mannose kinase
	B3GNT1	11q13.2	Synthesis of the linear poly-N-acetyllactosaminoglycans
	GMPPB	3p21.31	GDP-mannose pyrophosphorylase
Polymicrogyria			
	TUBB2	6p25	Major constituent of microtubules
	GPR56	16q21	G protein-coupled receptor 56
	SRPX2	Xq22.1	Plays a role in angiogenesis
	TBR2	3p24.1	Transcriptional activator
	PAX6	11p13	Transcription factor
	KIAA1279	10q22.1	Organization of axonal microtubules
	RAB3GAP1	2q21.3	RAB3 GTPase Activating Protein Subunit
Adams-Oliver syndrome (AOS)	ARHGAP31	3q13.33	Required for cell spreading
AOS	RBPJ	4p15.2	Plays a central role in Notch signaling
AOS	DOCK6	19p13.2	Atypical guanine nucleotide exchange factors
AOS	EOGT	3p14.1	EGF domain-specific GlcNAc transferase
AOS	NOTCH1	9q34.3	Play multiple roles during development
Heterotopia			
Heterotopia (X-linked Autosomal dominant)			
Classical bilateral periventricular heterotopia (PH)	FLNA	Xq28	Actin-binding protein
PH with fragile-X syndrome	FMR1	Xq27.3	Translation repressor
PH and Williams syndrome	WBSCR16	7q11.23	Guanine nucleotide exchange factor
PH	PVNH3	5p15.1	Periventricular Nodular Heterotopia 3
PH	PVNH5	5q14.3-q15	Periventricular Nodular Heterotopia 5
Heterotopia (Autosomal recessive)			
PH with microcephaly	ARFGEF2	20q13.13	Intracellular vesicular trafficking
PH with Donnai–Barrow syndrome	LRP2	2q31.1	Low density lipoprotein-related protein 2
Microcephaly			
	WDR62	19q13.12	Required for cerebral cortical development
	KIAA1279	10q22.1	Organization of axonal microtubules
	RAB3GAP1	2q21.3	RAB3 GTPase Activating Protein Subunit
	ARFGEF2	20q13.13	Intracellular vesicular trafficking
Focal cortical dysplasia			
	TSC1	9q34.13	Negatively regulating mTORC signaling
	TSC2	16p13.3	Negatively regulating mTORC signaling
Hemimegalencephaly			
	PIK3CA	3q26.32	Serine/threonine kinase - component of PI3K/AKT signaling
	AKT3	1q44	Serine/threonine kinase - component of PI3K/AKT signaling
	MTOR	1p36.22	Serine/threonine kinase – component of PI3K/AKT signaling

## Genes and brain malformations associated with defective neuron positioning

### Type I lissencephaly

Perhaps the best known type of neuronal migration disorder is lissencephaly, “smooth brain”. It is a brain malformation characterized by the absence of gyri and sulci [7, 33]. Most individuals with this condition also present with microcephaly (small head). Although the symptoms vary, they often feature seizures, intellectual disability, developmental delays, poor motor function, difficulties with feeding, and swelling in the extremities.

#### LIS1 and DCX

Mutations in *lissencephaly 1 (LIS1*) and *doublecortin* (*DCX*) have been shown to cause type I lissencephaly (Table 1). This disorder is often associated with axon outgrowth and guidance defects such as agenesis of the corpus callosum [34]. Neuronal positioning and further differentiation may coordinate to develop the pathogenesis of lissencephaly. Classic lissencephaly (type I) includes isolated lissencephaly and subcortical band heterotopia (“double cortex”) which are caused by *DCX* mutations [33, 35]. In addition, heterozygous mutation of *Lis1* in mice has been shown to impair normal neuron positioning and synaptogenesis in the amygdala [36]. Interestingly, there is a skewed sex ratio in subcortical band heterotopia and isolated lissencephaly. Females with a mutation affecting one copy of the *DCX* gene usually develop subcortical band heterotopia while males with one *DCX* gene mutation show isolated lissencephaly [37–41]. Males with subcortical band heterotopia or females with isolated lissencephaly are rarely reported [42, 43].

*DCX* encodes a microtubule-associated protein that stabilizes microtubules and causes bundling [44–46]. This is an important molecule in neuron migration and neurite growth in the developing brain [47–49]. *DCX* is expressed in neuronal precursor cells and immature neurons during brain development and in the adult hippocampus. More importantly, *DCX* is associated with the neuronal migration disorders, lissencephaly, pachygyria, and subcortical band heterotopia [37, 38, 41, 50–52]. Mutations in *DCX* prevent neurons from migrating into the cortical plate [45]. Abnormal microtubule functions dependent on *DCX* appear to underlie lissencephaly because pathological mutations in *DCX* prevent its product binding and subsequent stabilization of microtubules [53, 54].

Miller-Dieker syndrome is characterized as a congenital brain malformation due to the microdeletion of chromosome 17p13.3 including the *LIS1* gene, which can also cause classical lissencephaly [37, 41, 51, 55–58]. *LIS1* encodes a dynein-binding protein and controls mitotic spindle orientation in neural cells [59–61]. The most common type of mutation is a deletion of a single copy of the gene, resulting in haploinsufficiency. Individuals with *LIS1* mutations have not only lissencephaly, but often show other pathological features including corpus callosum hypoplasia and ventricle enlargement [58, 62]. These anatomical abnormalities correlate with the critical roles of *LIS1* in neuronal migration and axon formation [57, 58, 63]. In contrast to lissencephaly caused by mutations in *DCX*, *LIS1* mutations preferentially affect the parieto-occipital cortex [37, 41, 64]. Mutations in *LIS1* and *DCX* account for approximately 85% of patients with the classic form of lissencephaly [37, 41, 65].

#### YWHAE

*Tyrosine 3-Monooxygenase/Tryptophan 5-Monooxygenase Activation Protein, Epsilon* (*YWHAE*) is another gene that encodes a microtubule-associated protein and is located just 1 Mb away from *LIS1* on chromosome 17p. *YWHAE* also participates in the *LIS1* pathway, and homozygous deletion of mouse *Ywhae* leads to neuronal migration defects. Large deletions of the 17p13.3 region (which contains both *YWHAE* and *LIS1*) causes Miller-Dieker syndrome, and patients with this deletion display more severe neuronal migration defects than those observed in *LIS1* mutant heterozygote-caused lissencephaly [54].

#### TUBA1A and TUBB2

*Tubulin Alpha 1a* (*TUBA1A*) and *Tubulin Beta 2* (*TUBB2*) encode critical structural subunits of microtubules that are enriched during brain development [66]. *TUBA1A* mutations are identified in 1 % of classic lissencephaly and 30% of lissencephaly with cerebellar hypoplasia [67–69]. Meanwhile, *TUBB2* mutations are associated with symmetric polymicrogyria and pachygyria [70]. Guanosine triphosphate (GTP) contributes to microtubule assembly by binding to soluble tubulin heterodimers [71]. Mutations in these tubulin genes prevent microtubule polymerization. For example, the S140G mutation reduces the protein capacities of GTP binding and native heterodimer formation, thus preventing polymerization of microtubules and neuronal migration in mice [72]. In contrast to *TUBA1A* and *TUBB2*, *TUBB3* is important in axon guidance and microtubule dynamics, but dispensable for neuronal migration [73].

#### ARX

*Aristaless related homeobox* (*ARX*) is a homeobox-containing gene expressed in the nervous system during development [74–76]. *ARX* mutations are associated with an X-linked lissencephaly syndrome with infantile spasms as well as abnormal genitalia [77–79]. Mutations that cause lissencephaly often lead to premature truncation or alter the DNA binding domain of the protein (homeodomain) [80, 81]. Studies using human brain samples and animal models have revealed that *ARX* is important in proliferation of radial and intermediate neural progenitors, and migration of excitatory cortical neurons [75, 79, 80, 82]. It also critically controls the migration and further differentiation of inhibitory GABAergic interneurons [79, 80, 82–86]. This is consistent with the fact that ARX is expressed in the ganglionic eminence and cortical ventricular zone where interneuron and pyramidal neural progenitors reside, respectively [79]. Furthermore, ARX overexpression promotes the development of tangentially migrating interneurons [82, 86]. However, some mutations disrupt neuronal excitability without affecting neuronal migration or the cortical lamination pattern in the brain [87].

#### RELN

Reelin (RELN) and its cellular receptor very-low-density-lipoprotein receptor (VLDLR) are cellular signaling components. RELN is required for neuronal migration in the developing cortex [54, 88–91]. Accordingly, VLDLR critically regulates neuronal migration and positioning in the cerebral cortex [92]. RELN promotes hippocampal dendrite development through the VLDLR-Dab1 pathway as well [93]. Mutations in these genes are known to cause lissencephaly with cerebellar hypoplasia [54, 94–96]. The *RELN* mutation syndrome appears to be inherited in an autosomal-recessive pattern and these patients appear to be relatively rare [94]. Mutations in *VLDLR* can cause combinations of ataxia, intellectual disability, and quadrupedal gait [97].

### Type II lissencephaly

Type II lissencephaly is often referred to as “cobblestone lissencephaly” because patients typically only have regional agyria. It is associated with Walker-Warburg syndrome, a heterogeneous group of muscular dystrophy-dystroglycanopathy (MDDG) conditions that can be caused by homozygous mutations in the genes *FKTN* (Fukuyama syndrome), *POMT2* and *POMGnT1* (muscle-eye-brain disease), as well as *POMGNT2, FKRP, LARGE, ISPD, GTDC2, TMEM5, B3GALNT2, SGK196, B3GNT1,* and *GMPPB* [98–101]. In type II lissencephaly, there are no layers present in the cortex. Instead, irregularities in neuronal placement exist. Abnormal glycosylation of matrix proteins in the cerebral cortex is thought to cause these migration defects [102, 103].

Loss of function mutations in *SNAP29*, which encodes a member of the SNARE protein family, has been shown to cause CEDNIK (cerebral dysgenesis, neuropathy, ichthyosis and keratoderma) syndrome [104]. Brain MRI scans of CEDNIK syndrome patients revealed apparent extensive aberrant neuronal migration, as evidenced by corpus callosum abnormalities and cortical dysplasia, along with pachygyria, polymicrogyria and cobblestone lissencephaly [105]. Migration defects in *SNAP29* mutants may be attributed to an impairment in β1-integrin [106].

### Polymicrogyria

Polymicrogyria is a neurological condition characterized by an excessive number of small and fused gyri separated by shallow sulci in the cerebral cortex compared to normal cerebral surfaces [6, 70, 107, 108]. Mutations in the *TUBB2*, *GPR56*, and *WDR62* genes are associated with this condition [70, 109–111]. Polymicrogyria develops between the late stage of neuronal migration and the early point of cortical organization [108, 112]. Patients with polymicrogyria show a layer of intracortical laminar necrosis and subsequent disruption of late cortical lamination. Some cerebral cortices have a molecular layer that does not align along the borders of gyri. Neurons under this layer have a radial distribution without laminar organization [111]. Polymicrogyria most often occurs as an isolated feature. However, it is sometimes shown in multiple genetic syndromes associated with intellectual disability and birth defects including 22q11.2 deletion syndrome, Adams-Oliver syndrome (genetically heterogeneous, caused by mutations in *ARHGAP31, RBPJ, DOCK6, EOGT,* and *NOTCH1*), Aicardi syndrome, Galloway-Mowat syndrome, Joubert syndrome, and Zellweger spectrum (peroxisome biogenesis disorders including Zellweger syndrome, neonatal adrenoleukodystrophy, and Refsum disease) [111, 113–116]. The clinical features and etiology of polymicrogyria are heterogeneous. Most patients with polymicrogyria develop epilepsy during their early childhood (4–12 years of age). Seizures are resistant to pharmacological drugs in many cases of polymicrogyria.

#### *TBR2* and *PAX6*

*Pax6*, which encodes paired box protein 6, is highly expressed in radial glia, but is downregulated as they transition into intermediate progenitor cells during neurogenesis. This coincides with an upregulation of *T-brain gene-2 (TBR2*) that persists until intermediate neural progenitor cells differentiate into postmitotic neurons [117]. Mutations in *TBR2* and *PAX6* have been shown to cause polymicrogyria, due to defects in neuronal migration, differentiation and proliferation of neural progenitors [118–120].

#### SRPX2

*SRPX2* encodes a secreted sushi-repeat containing protein that is expressed in neurons. A rare missense mutation in the *SRPX2* gene causes bilateral perisylvian polymicrogyria, though its mechanism in development of this disease remains unknown. *SRPX2* is expressed in humans in the fetal and adult brain, whereas in mice, measurable expression does not begin until birth [121]. This poses problems for further studies into the role of *SRPX2* in brain development and neuronal migration.

#### KIAA1279

Homozygous nonsense mutations in the *KIAA1279* gene cause Goldberg-Shprintzen syndrome, which is characterized by bilateral generalized polymicrogyria, microcephaly, mental retardation, and an enteric nervous disorder [122]. *KIAA1279* encodes a kinesin family member-binding protein, but its role in the pathology of Goldberg-Shprintzen syndrome is still unknown [123, 124]. It was recently shown, however, that KIAA1279 co-localizes with both α-tubulin and F-actin. Relatedly, KIAA1279 is also involved in neurite outgrowth. Inhibition of KIAA1279 expression using siRNA leads to dendritic spine depletion and a decrease in neurite length in neuroblastoma cells, and overexpression of KIAA1279 triggers an increase in dendritic spine and neurite length, compared to controls [123].

#### RAB3GAP

Rab3 GTPase-activating protein (RAB3GAP) is a heterodimeric complex comprised of a catalytic subunit (RAB3GAP1) and a slightly larger non-catalytic subunit (RAB3GAP2). This complex acts as a guanine-nucleotide exchange factor for the RAB18 protein [125]. RAB18 is also regulated by the GTP-activating protein TBC1D20 [126]. Mutations or dysregulation of *RAB18* causes Warburg Micro syndrome, which is characterized by ocular and neurodevelopmental abnormalities, including polymicrogyria, microcephaly, pachygyria, polymicrogyria, and hypoplasia of the corpus calossum. It is unclear by what molecular mechanism RAB18 dysfunction leads to these neurodevelopmental aberrations, but mutations to *RAB3GAP1, RAB3GAP2, TBC1D20* and *RAB18* are all sufficient to cause these symptoms [125, 126]. It has recently been demonstrated, that TBC1D20 activity fosters extraction of RAB18 from the ER membrane and facilitates its retargeting for the *cis-*Golgi. In the *cis*-Golgi, it appears that the RAB3GAP complex recruits and stabilizes the RAB18 protein [126].

### Heterotopia

In addition to cortical gyration disorder, dysfunctional neuronal migration can lead to the development of neuronal population in aberrant locations. Periventricular nodular heterotopia is one of these neuronal migration disorders [5–7, 107, 108]. In this case, failed migration leads to the formation of heterotopic neurons along the ventricular surfaces in the brain. Therefore, the neurons are positioned deeper than those found in type I lissencephaly. This malformation can be bilateral or unilateral. Periventricular heterotopia is diagnosed with magnetic resonance imaging (MRI) and seizure symptoms. Affected individuals usually have normal intelligence, although some have mild intellectual disability. Some cases of periventricular heterotopia are associated with dyslexia [127]. For example, a specific reading fluency deficit is identified in a heterogeneous group of patients with periventricular heterotopia who have seizures, heterotopic neurons, and disrupted cortical connectivity [127, 128].

#### FLNA

The most common genetic cause of periventricular heterotopia is the X-linked dominant inheritance of *Filamin A* (*FLNA*) gene mutations [129, 130]. The *FLNA* gene encodes an F-actin-binding cytoplasmic protein involved in neurogenesis and neuronal migration in the developing brain [131, 132]. FLNA crosslinks actin filaments into the cortical cytoskeleton. *FLNA* mutations are associated with classical bilateral periventricular nodular heterotopia and account for the majority of X-linked inherited periventricular heterotopias [6, 133, 134]. *FLNA* regulates neuronal migration in the cerebral cortex [131]. Mutations in the human *FLNA* gene may also cause connective tissue disorders associated with Ehlers-Danlos syndrome which include extremely flexible joints, stretchable skin, and fragile blood vessels [135]. Unsurprisingly, patients with Ehlers-Danlos syndrome also frequently present with epilepsy and periventricular heterotopia [136].

#### PVNH3 and PVNH4

In addition to *FLNA* mutations, duplications and deletions in chromosome 5 which includes *Periventricular Nodular Heterotopia 3* (*PVNH3*) and *Periventricular Nodular Heterotopia 5* (*PVNH5*) have been seen in patients with periventricular heterotopia without mutations in other causative genes [137]. Periventricular nodular heterotopia is also found in individuals with other conditions, including Ehlers-Danlos syndrome [135].

#### FMR1

CGG trinucleotide repeat expansion of the *FMR1* gene causes fragile X syndrome in humans and has also been shown to lead to periventricular heterotopia. This may indicate a role for the FMR1 protein in neuronal migration [138].

#### ARFGEF2

*ADP-ribosylation factor guanine exchange factor 2 (ARFGEF2)* encodes a protein kinase A-anchoring protein that regulates GDP-GTP conversion of ADP-ribosylation factor [139, 140]. Via mediation of Filamin A signaling, *ARFGEF2* is involved in neuronal migration through the regulation of vesicle trafficking. Mutations in *ARFGEF2* also cause bilateral periventricular nodular heterotopia, as well as putaminal hyperintensity and microcephaly [131, 141].

#### LRP2

*Low density lipoprotein-related protein 2 (LRP2)* encodes megalin, a multiligand receptor. Mutations to *LRP2* cause Donnai-Barrow syndrome, which is associated with several neurological and cranial abnormalities, including periventricular nodular heterotopia [142]. Megalin facilitates the endocytosis of sonic hedgehog (Shh) in embryonic neuroepithelium [143]. Furthermore, megalin has been shown to bind and sequester Shh in the forebrain, and mediate Shh-Ptch endocytosis [144]. This key interaction with Shh signaling in the developing brain could explain the aberrant neuronal positioning observed in patients with *LRP2* mutations.

### Focal cortical dysplasia

Focal cortical dysplasia is a rare lamination abnormality in the cerebral cortex characterized by focal cortical thickening or thinning, focal atrophy, or blurring of the gray-white junction [6, 145]. Focal cortical dysplasia is the most common cause of medically refractory epilepsy in the pediatric population [145]. Defective regulation of neuronal migration or cell death is speculated to cause focal cortical dysplasia [146, 147]. There are three types of focal cortical dysplasia [34, 145, 148, 149]. Type I focal cortical dysplasia is found in the temporal lobe of the brain. This type is late onset, thus often seen in adults. Patients with this condition show mild symptoms. Type II focal cortical dysplasia, however, is mostly found in children and the clinical symptoms are more severe. There are more extensive changes outside the temporal lobe with predilection for the frontal lobes. Type III focal cortical dysplasia occurs in combination with hippocampal sclerosis, epilepsy-associated tumors, vascular malformation, or epileptogenic lesions. Studies have suggested that mutations in the *TSC1 *(*Tuberous Sclerosis 1*) gene is associated with the formation of focal dysplasia [145, 150, 151]. Changes in Wnt and Notch signaling components that control proper neuronal migration are also found in focal cortical dysplasia [145, 152].

### Hemimegalencephaly

Hemimegalencephaly is implicated in neuronal positioning abnormality. Hemimegalencephaly features one side of the brain that is abnormally larger than the other [6, 108, 153]. The unusual enlargement of the brain causes seizures and intellectual disability [154]. This condition is thought to take place when neurons are abnormally organized due to defective migration in the developing cerebral cortex because the enlarged hemisphere usually shows focal or diffused regions of polymicrogyria, pachygyria, and heterotopia [155–159]. However, whether abnormal neuronal migration during development causes hemimegalencephaly is unclear. Using exome sequencing, recent studies have identified *de novo* germline and somatic mutations of PI3K-AKT-mTOR components (PIK3CA, AKT3, and MTOR genes) in patients with hemimegalencephaly [160–164]. Thus, hemimegalencephaly may be a genetically mosaic disease caused by abnormal PI3K-AKT-mTOR signaling. In addition to its role in neuronal migration, PI3K-AKT-mTOR signaling critically regulates neural progenitor proliferation and neurogenesis [32, 165–168].

## Conclusions

Recent advances in neurogenetics and brain imaging have revealed genes responsible for neuronal migration disorders. Efforts have been made to characterize the functions of the causative genes and develop appropriate animal models. Still, research that overcomes these disorders is only in the beginning stage of work. Further human genetic analysis and neurobiological studies should expand our understanding of the pathogenesis of neuronal migration disorders, which will help to develop therapeutic strategies for these disorders in the future.

## Abbreviations

AKT: RAC-alpha serine/threonine-protein kinase
ARGEF2: ADP-ribosylation factor guanine exchange factor 2
ARHGAP31: rho GTPase Activating Protein 31
ARX: Aristaless-related homeobox
B3GALNT2: Beta-1,3-N-acetylgalactosaminyltransferase 2
B3GNT1: Beta-1,3-N-acetylglucosaminyltransferase 1
CEDNIK: Cerebral dysgenesis-neuropathy-ichthyosis-palmoplantar keratoderma
CP: Cortical plate
DCX: Doublecortin
DOCK6: Dedicator of cytokinesis 6
EOGT: EGF domain-specific O-linked N-Acetylglucosamine (GlcNAc) transferase
FKRP: Fukutin related protein
FKTN: Fukutin
FLNA: Filamin A
FMR1: Fragile X mental retardation 1
GMPPB: GDP-mannose pyrophosphorylase B
GPR56: G protein-coupled receptor 56
GTDC2: Glycosyltransferase-like domain containing 2
GTP: Guanosine triphosphate
ILS: Isolated lissencephaly sequence
ISPD: Isoprenoid synthase domain containing
IZ: Intermediate zone
KIAA1279: KIF1 binding protein (KIF1BP)
LARGE: Like-glycosyltransferase
LCH: Lissencephaly with cerebellar hypoplasia
LGE: Lateral ganglionic eminence
LIS1: Lissencephaly 1
LRP2: Low density lipoprotein receptor-related protein 2
MEB: Muscle-eye-brain disease
MRI: Magnetic resonance imaging
mTOR: Mechanistic target of rapamycin
MGE: Medial ganglionic eminence
MZ: Marginal zone
PAX6: Paired box 6
PH: Periventricular heterotopia
PI3K: Phosphatidylinositol-4, 5-bisphosphate 3-kinase
POMGnT1: Protein O-linked mannose N-acetylglucosaminyltransferase 1
POMGnT2: Protein O-linked mannose N-acetylglucosaminyltransferase 2
POMT2: Protein-O-mannosyltransferase 2
PVNH3: Periventricular nodular heterotopia 3
PVNH4: Periventricular nodular heterotopia 4
RAB18: RAB18, member RAS oncogene family
RAB3GAP: Rab3 GTPase activating protein
RBPJ: Recombination signal binding protein for immunoglobulin kappa J region
RELN: Reelin
RGP: Radial glial progenitors
RMS: Rostral migratory stream
SBH: Subcortical band heterotopia
SGK196: Protein-O-mannose kinase (POMK)
SNAP29: Synaptosomal-associated protein, 29kDa
SNARE: Soluble NSF Attachment Protein Receptor
SRPX2: Sushi-repeat containing protein, X-linked 2
SVZ: Subventricular zone
TBC1D20: TBC1 domain family, member 20
TBR2: T-brain gene-2
TMEM5: Transmembrane protein 5
TSC1: Tuberous sclerosis 1
TSC2: Tuberous sclerosis 2
TUBA1A: Tubulin, alpha 1a
TUBB2: Tubulin, beta 2
TUBB3: Tubulin, beta 3
VLDR: Very-low-density-lipoprotein receptor
VZ: Ventricular zone
WBSCR16: Williams-Beuren syndrome chromosome region 16
WDR62: WD repeat domain 62
WWS: Walker-Warburg syndrome
YWHAE: Tyrosine 3-monooxygenase/Tryptophan 5-Monooxygenase Activation Protein, Epsilon
